# Evaluating the Biodegradation of Veterinary Antibiotics Using Kinetics Model and Response Surface Methodology

**DOI:** 10.3390/molecules27175402

**Published:** 2022-08-24

**Authors:** Martha Noro Chollom, Babatunde Femi Bakare, Sudesh Rathilal, Emmanuel Kweinor Tetteh

**Affiliations:** 1 Environmental Pollution and Remediation Research Group, Department of Chemical Engineering, Mangosuthu University of Technology, P.O. Box 12363, Durban 4026, South Africa; 2 Gree Green Engineering Research Group, Department of Chemical Engineering, Faculty of Engineering and the Built Environment, Durban University of Technology, Steve Biko Campus, S4 Level 1, Durban 4000, South Africa

**Keywords:** anaerobic digestion, antibiotics, biodegradation, ciprofloxacin, enrofloxacin, response surface methodology, kinetics, wastewater

## Abstract

The inappropriate use and indiscriminate disposal of antibiotics has become a menace worldwide. The incomplete removal of these contaminants from wastewater treatment plants has also contributed to this. This study presents the biodegradation of two veterinary antibiotics; ciprofloxacin (CIP) and enrofloxacin (ENRO). Kinetics models were explored to understand the dynamics of biodegradation in an anaerobic digestion process. This was carried out in batch reactors under various operating conditions: pH, organic loading rate (OLR), and antibiotic concentration. The influence of the parameters was investigated using a response surface methodology (RSM) based on the Box–Behnken experimental design of 15 runs. The data obtained were fitted on a polynomial function model. OLR and pH exhibited a synergistic and antagonistic effect in the response models developed, with a high correlation regression coefficient (R^2^; 0.9834–0.9875) close to 1 at a 95% confidence level. The optimum conditions obtained from the RSM numerical optimization were pH (6), OLR (2 kgCOD·m^−3^·days^−1^), and an antibiotic concentration of 75%, which gave the removal of CIP, ENRO, and COD, respectively, as 80%, 83%, and 73% at a desirability function of 85%. The kinetics study shows that the biodegradation of antibiotics was well fitted on a first-order model (R^2^; 0.9885–0.9978) with rate constants ranging from 0.0695 to 0.96 days^−1^.

## 1. Introduction

Pharmaceuticals are emerging environmental contaminants, which are extensively used for human and veterinary medicine [1]. Antibiotics represents about 70% of all the consumed pharmaceuticals that are used for human and animal medicines, usually as a therapy or curative to enhance life expectancy. With regards to veterinary antibiotics (ABs), they are used widely to promote animal growth, especially in practices where the animals are confined in large numbers [2,3]. The consumption of antibiotics has remained stable in high income countries, while for developing countries, a steady increase is observed, especially with population growth [3,4]. Thus, millions of tons of antibiotics are produced globally on a yearly basis.

These antibiotics contain complex organic compounds in their structures, thus making their degradation to simpler products difficult such that they can persist and bioaccumulate in the aquatic environments for long periods. This is due to the fact that not all of the antibiotics taken for therapeutic and curative purposes are completely absorbed, but only about 20–30% are absorbed in some cases. The rest (active substances) are ejected from the body (in the urine and feces) into the environment and wastewater treatment plants without complete metabolization. Thus, the wastewater treatment plants and hospitals, amongst others, are the main sources through which these contaminants are released into the environment [1]. Due to the hydrophobicity and lipophilic nature of ABs and their residues, their elimination from wastewater becomes difficult. In addition, their persistence in the environment is greatly prompted by their physicochemical properties which include low hydrosolubility, low log P (log of the partition coefficient octanol/water), and their being amphoteric.

The appearance of antibiotics in the environment has been linked to an increase in the number of resistant bacteria. The rapid growth of drug-resistant infections is alarming, it is estimated that by 2050 this will lead to 70,000 deaths per year [1,3]. This has economic consequences, therefore, proactive actions including engineered technology and waste management should be improved [4].

Studies on antibiotics have drawn much attention due to the high detection frequency of antibiotics in the environment [1,3,4,5]. The detection of antibiotics in the environment is found to be in the range of ng/L and µg/L, and even mg/L [6,7].

Various classes of antibiotics exist such as the β-lactams, fluoroquinolones, tetracyclines, and sulphonamide. There are a few criteria required for the prioritization of these antibiotics, some of which are: (1) the relevance of antibiotics to human and animal usage, (2) usage amongst the different animal species, and (3) their detection in wastewater treatment plants and the environment [8,9]. For instance, fluoroquinolones (FQs) are the third group of antibiotics that are commonly used worldwide. They have about 17% of the global market share value. This is due to the fact that they are used both in animals and humans. Detection levels in wastewater treatment sludge range from 2 to 510 µg/L, while it is 10 to 250 ng/L in surface waters. Reported concentrations from manures and wastewater bodies released from livestock farms are reported to be within the range of 1.4 to 5.3 µg/L and 63 to 585 ng/L, respectively [7,10].

The removal of antibiotics from wastewaters can be achieved using biotic and abiotic processes. The biotic process involves biodegradation by microorganisms, while the abiotic processes are sorption, hydrolysis, oxidation-reduction, and photolysis [11]. Even though chemical, biological, and advanced oxidation processes have shown capabilities in eliminating organic contaminants and other derivatives, there is still limited knowledge on effective removal of ABs and other emerging contaminants using anaerobic digestion (AD) process, since some of them are bio-recalcitrant [1,4,12]. Therefore, this study investigated the biodegradation dynamics of two veterinary antibiotics viz. ciprofloxacin (CIP) and enrofloxacin (ENRO) from industrial wastewater via anaerobic digestion. The investigation was carried out in batch reactors under a varying pH, organic loading rate (OLR), and antibiotic concentration. The influence of the aforementioned parameters was then investigated with kinetics models and response surface methodology to understand their interaction during anaerobic digestion.

## 2. Results

### 2.1. Biodegradation

#### 2.1.1. COD Removal

**Figure 1:**
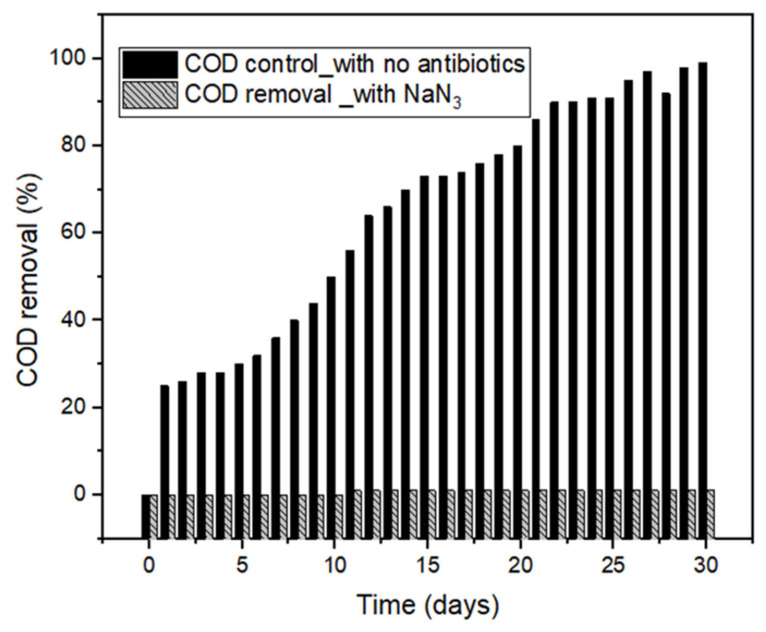
Biodegradation effect on COD removal in control reactors at 35 °C for 30 days 35 °C, pH 7, and OLR at 3.5 kg COD·m^−3^·days^−1^.

**Figure 2:**
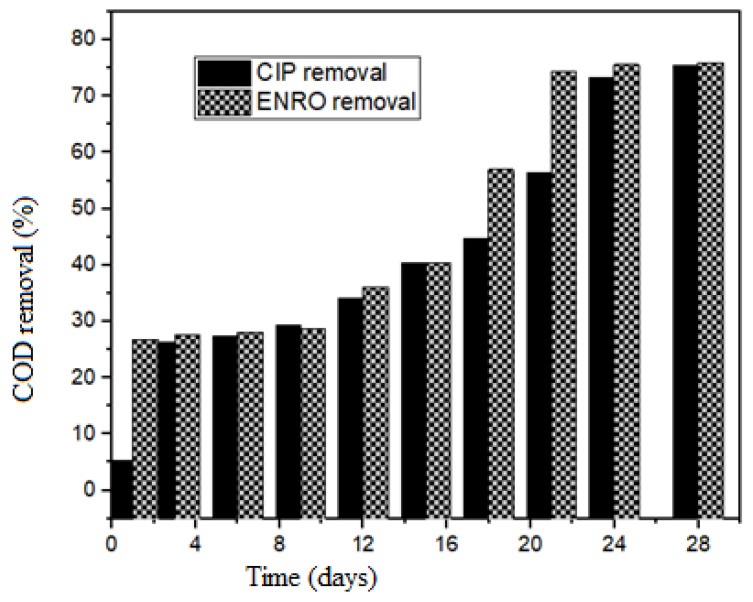
COD removal during biodegradation of wastewater at 35 °C, pH 7, and OLR at 3.5 kg·CODm^−3^·days^−1^.

The biodegradation experiment was carried out at a pH of 6.8, OLR of 3.5 kgCOD·m^−3^·days^−1^, temperature of 35 °C, and initial antibiotics concentration of 100 ug/L. The biodegradation experiment was carried out to ensure that that the dosage of antibiotics added to the bioreactors did not inhibit the microorganisms. The overdosing of the antibiotics could affect their performances. The bioreactors consisted of two control bioreactors, the first one had no antibiotics while in the second bottle, sodium azide (NaN_3_), was added to the reactor to inhibit the microbial activity. Figure 1 shows the COD removal of the bioreactors for a period of 30 days. From Figure 1, it can be seen that there was no significant removal of COD in the reactor that contained the NaN_3_, thus indicating that NaN_3_ had inhibited microbial activities. However, COD removal increased in the control reactor with no antibiotics added to it. Figure 2 shows COD removal in the reactors containing antibiotics (CIP and ENRO) at a concentration of 100 µg/L. This was the highest concentration of the antibiotics used. Comparing the COD removal in the reactors with the antibiotics (Figure 2) and control (Figure 1), an increase in COD the removal was observed. This could have been mainly because antibiotics inhibit AD by affecting microbial metabolism, growth, and reproduction.

#### 2.1.2. Antibiotics Removal

**Figure 3:**
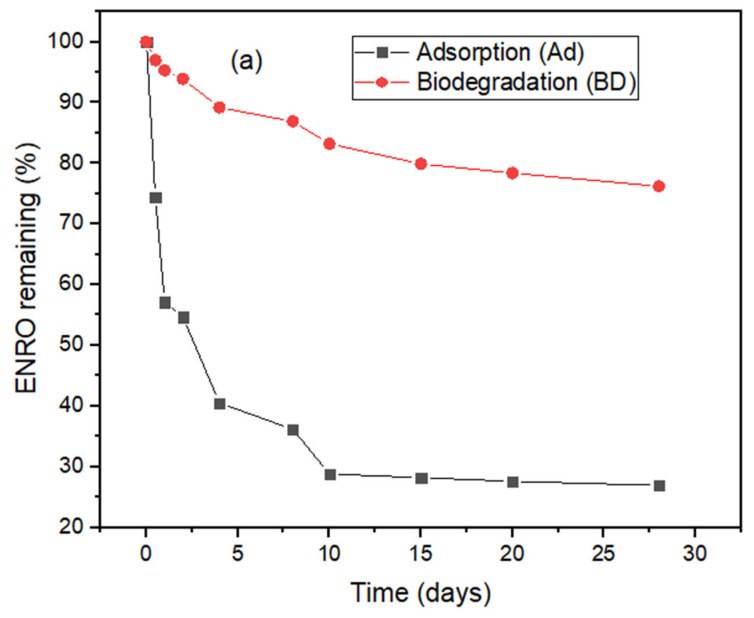

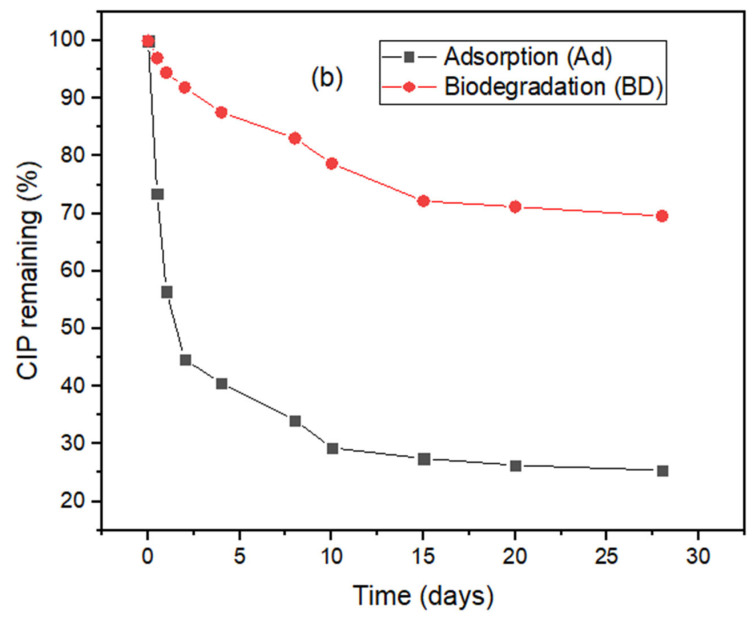
Biodegradation (BD) and Adsorption (AD) of (**a**) %ENRO and (**b**) %CIP removal at 35 °C, pH 7, and OLR at 3.5 kg·CODm^−3^·days^−1^.

The removal of antibiotics during a biological process such as anaerobic digestion could be mainly through adsorption (Ad) and biodegradation (BD), others, such as hydrolysis and volatilization, are negligible. For this experiment, both adsorption and biodegradation was observed. Figure 3 shows the removal of the antibiotics (CIP and ENRO) during biodegradation. From Figure 3, it was observed that the percent removal went from 100 to less than 50% in 3 days. It was comparatively fast in the early stages of the AD system as compared to the BD system. This could have been because the antibiotics affected the process negatively, which remarkably showed a 20–30% biodegradability rate, as reported by [13], and also due to the adsorption of antibiotics to the sludge. Further descriptions of adsorption were found in previous work [14]. The authors used batch reactors and continuous anaerobic reactors to evaluate the removal routes of the five different classes of antibiotic. They observed that both the adsorption and biodegradation removal of antibiotics exists in an AD process. However, the removal pathway of each class depended on the nature of the antibiotics.

Rapid adsorption was observed in the first few days and only 25% of the ENRO remained after 10 days. There was little further adsorption between day 10 and day 28.

The high removal at the end of 28 days indicated that both biodegradation and adsorption were happening, thus reducing the amount antibiotics in the system. Various researchers have reported that the removal of antibiotics during biological processes is divided into two: adsorption and biodegradation. In the first stage, adsorption occurs faster than biodegradation. At this stage, biodegradation is limited due to the fact that microorganisms are still adapting, hence they take time to be fully active. The second stage is mainly biodegradation. At this stage, the migration and biotransformation of the antibiotics to the surface of the sludge takes place, hence improving degradation [1].

#### 2.1.3. Kinetics Studies

**Figure 4:**
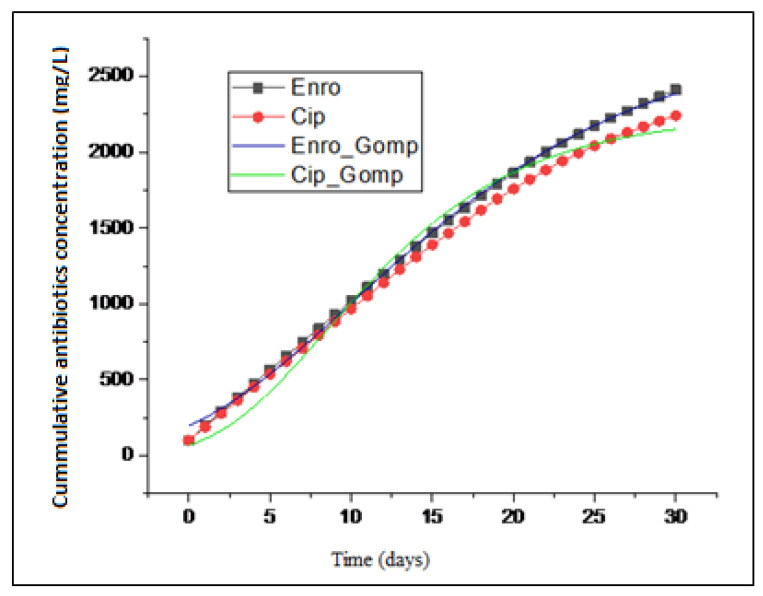
Data fitting of ENRO and CIP biodegradation to Gompertz model.

**Figure 5:**
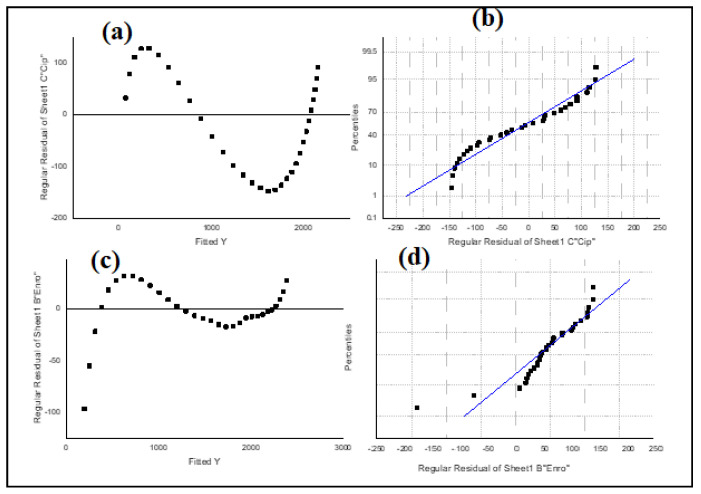
Gompertz model patterns. (**a**) CIP residual plot, (**b**) CIP line of best fit, (**c**) ENRO residual plot, (**d**) ENRO line of best fit.

A modified sigmoidal bacterial growth curve (Gompertz) expressed in Equation (1) was fitted (Figure 4) with the data obtained using OriginPro software (version 2019; OriginLab, Northampton, MA, USA). Figure 5 describes the degradation patterns associated with the complex substrates composing the antibiotics. To ascertain the best fit, the goal was to minimize the sum of the square differences between the predicted and the measured values. In addition, the correlation coefficient (R^2^) was determined at a 95% confidence interval to predict the goodness of fit. Using the analysis of variance (ANOVA), the minimum sum of the squares and model constants obtained are presented in Table 1. To determine the apparent elimination rate constant and half-life for the biodegradation, the half-lives of the compounds t^1/2^ were calculated from the equation to give $t_{1/2}\frac{\left(\ln2\right)}{K_{1}}$. Half-lives of 14 and 10 days were obtained for both ENRO and CIP.

(1) $$\frac{dC}{dt}=-k\cdot C\Leftrightarrow C_{t\;}=C_{0}\cdot e^{-k\cdot t}$$

where $C_{0}$ is the initial concentration of the antibiotic, and $C_{t}$ is the concentration of the antibiotic at a specific time (*t*) and rate constant $\left(k\right)$.

The half-lives of various pharmaceuticals have been reported. For example, in the review by Bavumiragira et al. [11], they reported that that diclofenac, bezafibrate, ibuprofen, naproxen, and gemfibrozil were significantly biodegraded, possessing half-lives of 2.5–18.6 days in waters with suspended sediments under aerobic conditions. The biodegradation in the surface water for ciprofloxacin was found to be 376 h (approximately 16 days).

### 2.2. Response Surface Methodology

As stated earlier, the influence of the process variables (pH, OLR, and **ANT**) on the antibiotics’ degradation was investigated by using single component techniques. To further understand their interactive effect on the antibiotic’s removal, the design range and levels of the three process parameters were then statistically optimized by the RSM. The experiment was randomly performed according to the experimental design matrix of the Box Benhken Design (BBD) adapted from the RSM (Table 2). A total of 15 experiments with different combination levels of pH, OLR, and ANT concentration showed significant effects on the CIP, ENRO, and COD removal. The results presented in Table 2 show the observed and predicted responses with considerable variations in the antibiotic’s biodegradation activity. The experimental runs (Table 2) had three duplicated center points (runs 3, 5, and 9) that were meant for the estimation of the pure error sum of the squares by the response model developed. It was observed that the degradability of the CIP, ENRO, and COD was within 65–90%, with a significant deviation between the predicted and observed data. To evaluate the relationship between the dependent and independent variables as well as maximize the degradation efficiency concerning optimum conditions, the second order model (2) was proposed to calculate the optimum levels of the input variables. By applying the analysis of variance (ANOVA) and regression fit analysis, a second-order polynomial function expressed in (2) was used to explain the role of each model term and their interactive effect on the response.

(2) $$Y=\beta_{0}+\overset{n}{\underset{i=1}{\sum }}\beta_{i}X_{i}+\overset{n}{\underset{i=1}{\sum }}\beta_{\mathrm{ii}}X_{\mathrm{ii}}^{2}+\overset{n}{\underset{i<j}{\sum }}\beta_{\mathrm{ij}}{\;X}_{i}X_{j}+ɛ$$

where the terms *Y*, $\beta_{0},\;\beta_{i},\beta_{ii},\;\beta_{ij}$, and ɛ represent the response, independent variable effects, linear coefficients, squared coefficients, interaction coefficients, and the intercept.

#### 2.2.1. Response Model

Different regression model analyses were performed at a 95% confidence level using multiple model selection methods and criteria with *p*-values < 0. 05. The ultimate models developed for each response are expressed in (3) to (5) with each term in coded form. These are the reduced quadratic models (3) and (4) and two-factor interaction (5) terms obtained after the neglection of the insignificant terms (based on the *p*-values which were greater than 0.5). The significance of the models corresponds to the magnitude of the estimated coefficient and standard errors. This implies that the linear, quadratic, and interactive effects of the pH (A) and OLR (B) on the responses were more significant than the others. Thus, the pH and OLR had a direct relationship to the levels of the antibiotic’s concentration. This suggests that a variation in the antibiotic’s concentration altered the biodegradation rate of the wastewater.

(3) $$\begin{array}{c} CIP=-9.81+21.056A+0.583B+0.425C+0.125AB-0.0389AC\\ \;\;\;\;\;\;+\left(8.33\times 10^{-3}\right)BC-1.4583A^{2}-0.2396B^{2}\\ \;\;\;\;\;\;-\left(2.263\times 10^{-4}\right)C^{2} \end{array}$$

(4) $$\begin{array}{c} \mathrm{ENRO}\;removal=87.083-3.0417A-11.528B+0.425C+1.625AB\\ \;\;\;\;\;\;-0.0333AC+\left(2.778\times 10^{-3}\right)BC \end{array}$$

(5) $$\begin{array}{c} COD\;removal=112.083-5.778A-3.9514B-0.157C+0.5AB+0.0111AC\\ \;\;\;\;\;\;-\left(2.778\times 10^{-3}\right)BC+0.20833A^{2}+0.1146B^{2}\\ \;\;\;\;\;\;-\left(5.144\times 10^{-4}\right)C^{2} \end{array}$$

The coefficient sign of each model term demonstrated the significance and effects model developed as shown in Table 3.

Thus, a negative or positive sign before each term represents antagonistic or synergistic effects of the term on the antibiotics’ degradation efficiency, respectively. Among the variables, the two-factor interactional effects were also investigated. This was done by fixing one factor at its high (+1) or low (1) level, and establishing the significant effect of the other two variables on the response. It was found that the interaction between pH (*A*) and OLR *(B*) had a significant effect on all the responses. In addition, the interactional effect between pH (*A*) and antibiotic concentration (*C*) had less significant effects on the degradation of *CIP* and ENRO at a low OLR. The ranking order of the two interactional variables such as *AB* > *BC* > *AC* was found to increase the removal of CIP and ENRO, whereas *AB* > *AC* > *BC* significantly contributed to the reduction of *COD*. However, increasing each response based on the significant effects of the independent variables is expressed as *A* > *B* > *C* for *CIP*, *C* > *A* > *B* for ENRO, and *C* > *B* > *A* for *COD* removal.

#### 2.2.2. Analysis of Variance (ANOVA)

The significance and adequacy of the models were determined by the ANOVA, whereas the model fitness was verified by the correlation coefficient R^2^ of the model and *p*-value for the lack of fit. Table 4 shows that the smaller the value of (Prob > F), the more significant the corresponding model term. Usually, the value of (Prob > F) over 0.1 implies that the model term is insignificant, and terms such as these are excluded to improve the precision of the model prediction. The determination coefficients (R^2^) of the regression models obtained were 0.9834; 0.9827 and 0.9875 for CIP, ENRO, and COD, respectively.

The coefficient of variation (CV) was then applied to articulate the reason for the high R^2^ values of the models, as well as to determine the extent to which the data were dispersed. The CV is a measured lack of fit (LOF) between the predicted and experimental data, which was calculated using the difference between the sum of the squares of the response variable and its predicted values by the model [15]. In Table 4, the CV values of 1.63%, 2.48%, and 1.73%, respectively, for the CIP, ENRO, and COD models were within the acceptable range (0.5–13.5%), which makes the models adaptable. The standard deviation (1.24, 1.89, and 1.3) as a percentage of the mean (76.13, 76.33, and 75.13) concerning the CIP, ENRO, and COD responses are also presented in Table 4. Adequate precision was used to measure the signal-to-noise ratio. The values of adequate precision greater than four were desirable. Additionally, the adequate precision values of 17.8, 16.7, and 13.8 for CIP, ENRO, and COD, respectively, denoted adequate signals for the models to be used to navigate the design space.

**Figure 6:**
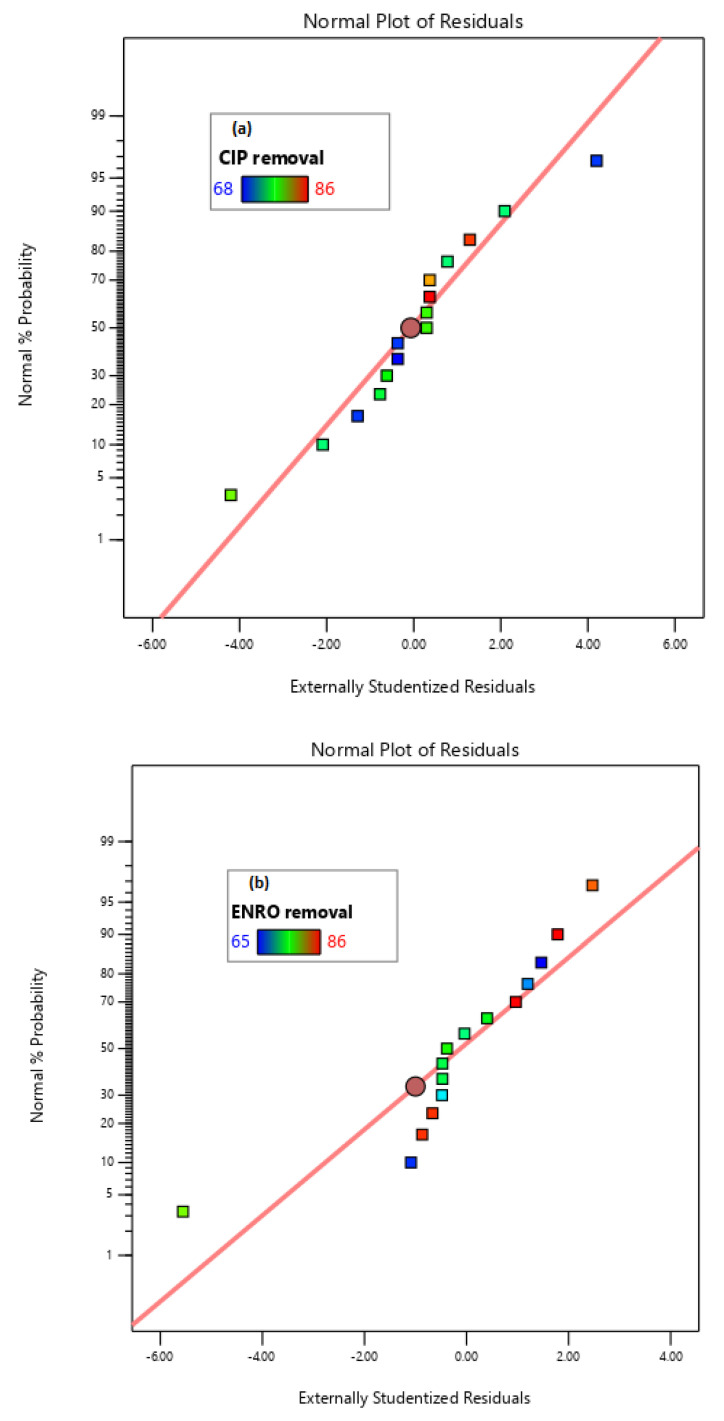

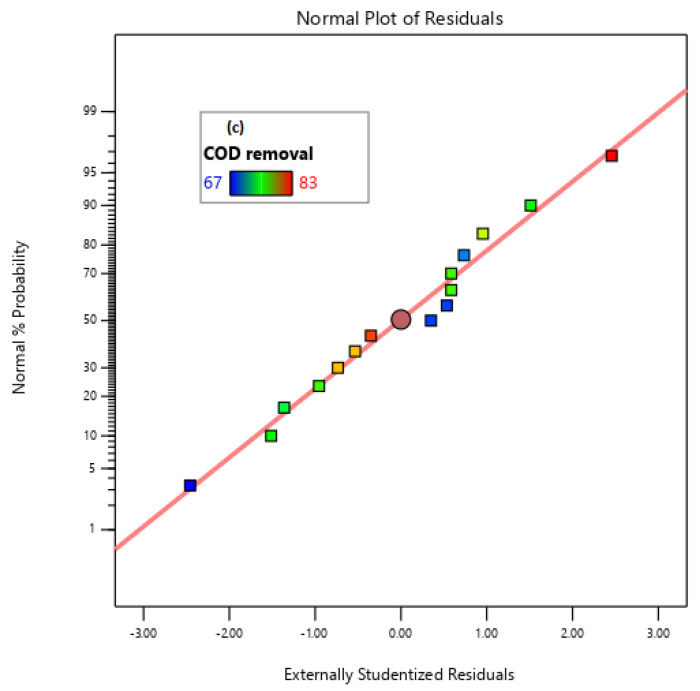
Normality plot of (**a**) CIP, (**b**) ENRO, and (**c**) COD removal.

The graphical representation using the RSM heightens the visualization of the interactive effects of the models developed. As shown in Figure 6, the normal residuals fall along a straight line, indicating that there is no apparent problem with normality and no need for the transformation of the response removal efficiency within the 65–86%. The normal probability plots showed that all the residuals follow a normal distribution, which was fitted diagnostically.

**Figure 7:**
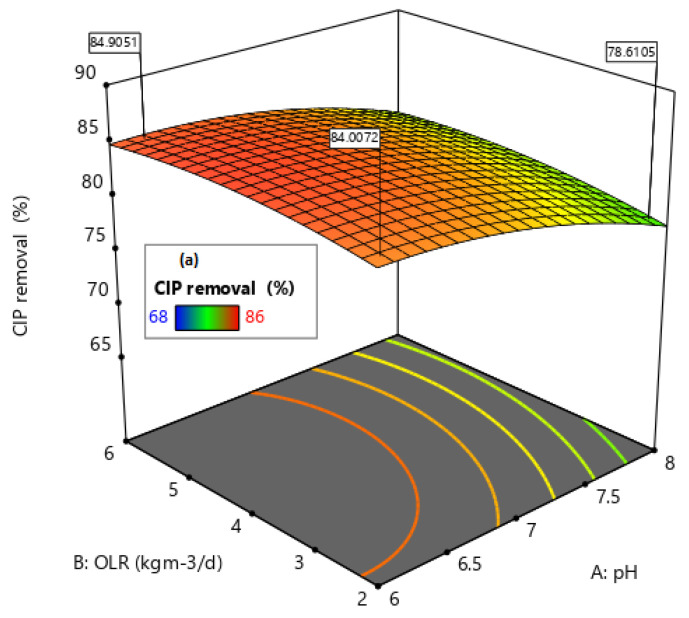

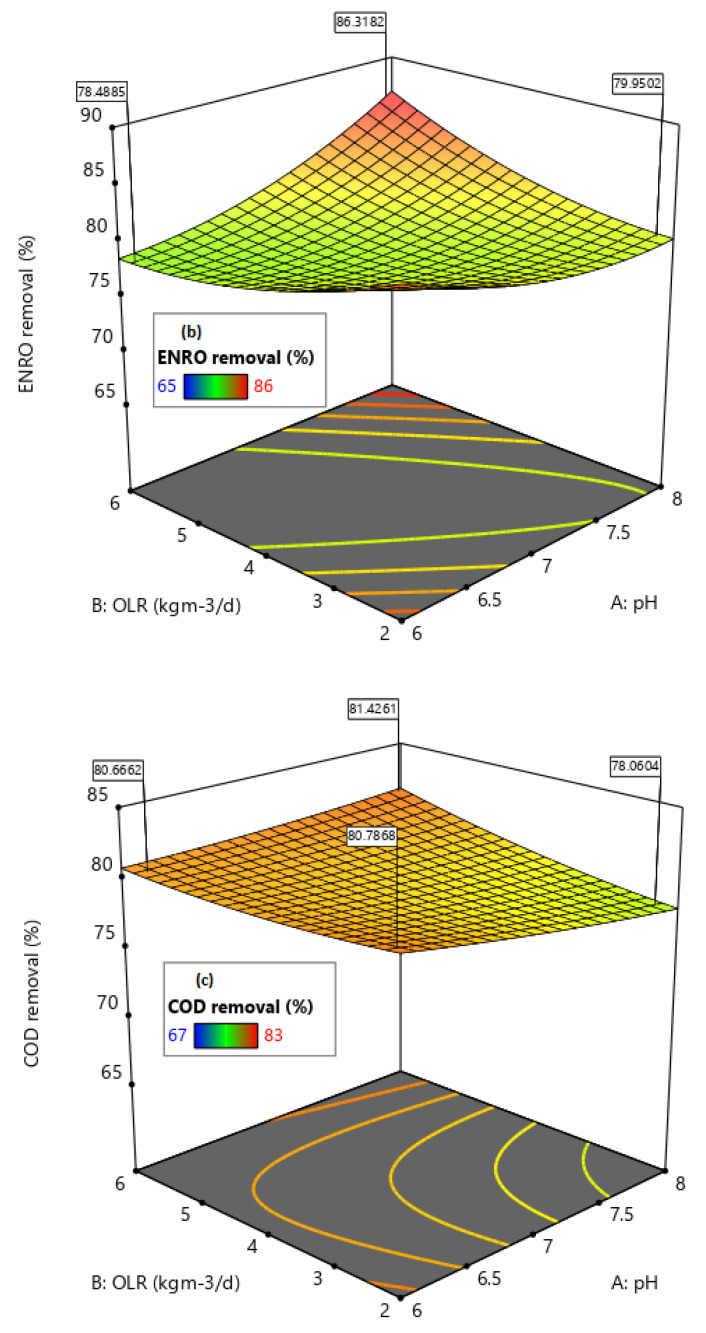
Response 3D plot of pH and OLR effect at constant antibiotic concentration on (**a**) CIP, (**b**) ENRO, and (**c**) COD % removal.

The response removal was graphically shown through three-dimensional (3D) plots. The effect of pH (A) and OLR (B) on CIP, ENRO, and COD removal in relating to their respective model Equations (4)–(6) are presented in Figure 7.

**Figure 8:**
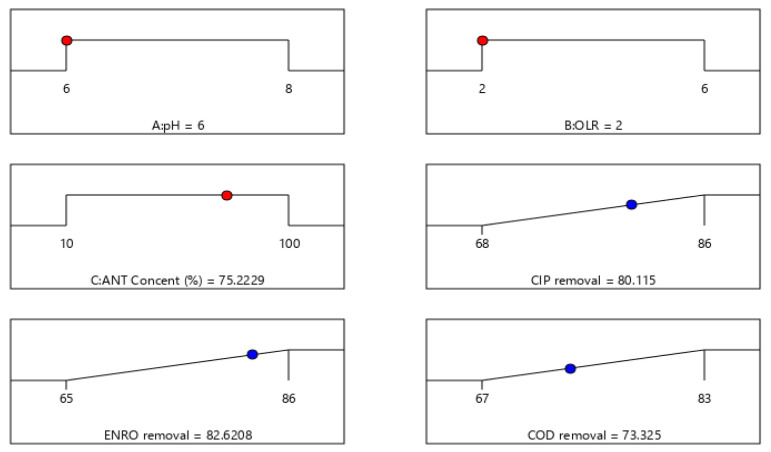
Ramp plot of optimum conditions and desirable responses.

Optimization using the desirability function was employed to determine the optimum conditions for the maximum removal of CIP, ENRO, and COD. The possible optimizations input that can be selected include the range, maximum, minimum, target, or none for the response. In this context, the input variables were specified within range values, whereas the responses were designed to achieve maximum at a 95% confidence level. Using these conditions, Figure 8 shows the maximum removal of CIP (80%), ENRO (83%), and COD (73%) at optimum conditions of pH (6), OLR (2 kgCOD·m^−3^·days^−1^), and an antibiotic concentration of 75%.

## 3. Discussion

### 3.1. Biodegradation and Kinetics

In this study, operating parameters in bioreactors for the removal of antibiotics from the wastewater were employed. This was carried out in the anaerobic digestion process, whereby the hydrolysis, acidogenesis, and methanogenic activities of the microorganism contributed to the catabolic and anabolic utilization of the antibiotics and organic substrates. Therefore, to understand the effect of operating conditions such as pH, OLR, and the addition of antibiotics on the performance of the bioreactors, the response surface methodology (RSM) and kinetic models with the experimental matrix presented in Table 2 was used. As shown in Table 5, although CIP and ENRO are of the same class, their molecular structure differs. This generates active chemical species, which undergo a series of chain reactions to form by-products [16]. In addition, the antibiotics are biotransformed, yielding less active metabolites.

Residual plots in Figure 1 show that there is a gradual degradation of the antibiotics (ENRO and CIP) to a point that the microbes become harmonized with the antibiotics to enhance their metabolism. This might be due to the antibiotics facilitating the active site to be inhibitory to the enzyme or microorganism [6,14]. The assumption on biodegradation that antibiotics do not degrade extensively under anaerobic conditions was confirmed (Figure 2) [7,14]. Figure 3 shows that the removal of fluoroquinolones (ENRO and CIP) is more recalcitrant during biodegradation. This is due to their amphoteric characteristics and the octanol–water partition coefficient. For example, Zhang and Li [17] found that the biodegradation of norfloxacin and ofloxacin in the anaerobic digestion systems was weaker than that of tetracyclines, sulphonamides, and macrolides.

Figure 4 and Figure 5 reveal that both antibiotics followed the first order degradation, with coefficients of correlations close to 1. Table 1 presents the values for the minimum sum of the square (194.46; 576.23), R^2^ (0.9978; 0.9885), and k (0.096; 0.0695), respectively, for ENRO and CIP obtained at a half-life of 14 and 10 days, respectively. The rate constant (k) expression described the kinetics of the microbial activity on the antibiotics, which is in accordance with the report in [18]. It was deduced that the growth rate of the microorganisms is proportional to the rate of substrate utilization. Therefore, the current assumption that antibiotics do not degrade completely under anaerobic conditions is correct, rather, the biodegradation occurred alongside adsorption, even though it was with a lower degree. A similar observation was made by Li and Zhang [19]. The removal of pharmaceuticals, including antibiotics, using various mechanisms, such as constructed wetland (CW) and stabilization ponds (SP), lead to two conventional wastewater treatment processes (activated sludge (AS) and micro-power biofilm (MP) [4,20]). They were able to detect antibiotics in both the influent and effluent samples. Their findings generally showed an incomplete removal of antibiotics with detection frequency variation. This study also supports the previous research that showed how antibiotics vary in water settings due to the nature of the antibiotics, as well as the source of the wastewater.

Previously, the inhibition of the anaerobic processes has been of heavy metals such as Ni > Ca > Pb > Cr > Zn with iron. These metals, in varying concentrations of (mg/L), are said to inhibit the anaerobic process. On other hand, inhibitory substances studied for anaerobic digestion processes have always focused on the decreasing toxicity of the heavy metals such as Ni > Ca > Pb > Cr > Zn with iron, and are considered more beneficial than detrimental because of its mediating effects on sulfide toxicity [21,22]. Chollom et al. [14] characterized influent and effluent samples from a local South African slaughterhouse wastewater treatment plant. Among the veterinary antibiotics detected, fluoroquinolones (CIP and ENRO) were within 9.1–10.6 ng/L in the effluent. This has now become very crucial because studies have indicated the inability of the anaerobic digestion systems to eliminate this bio-recalcitrant contaminant [14].

### 3.2. Response Surface Methodology

The impact of the three independent variables (pH (A), OLR (B), and antibiotics concentration (C)) were studied on CIP, ENRO, and COD removal in the form of experimental and predicted responses. Among the input variables coefficients, pH was found to have the greatest effect with a positive impact for the removal of CIP, whereas ENRO and COD removal were negatives (3–5).

Thus, pH had a synergistic effect on the degradation of CIP and an antagonistic effect on ENRO and COD due to their physicochemical properties. Li and Zhang [19], and Alqarni et al. [1] reported that very high pH values lead to increasing HO_2_ and the consumption of OH radicals, contributing to the neutrality of the antibiotics’ charge surface, thereby reducing the degradation.

The significance and adequacy of the models were determined by the ANOVA (Table 3 and Table 4), whereas the model fitness were verified by the correlation coefficient R^2^ of the model and *p*-value for lack of fit (LOF). The R^2^ value is a measure of discrepancy in the response values, which are based on the experimental variables and their interaction effects (pH and OLR) [31]. The Predicted R^2^ values were also found to be in reasonable agreement with the adjusted R^2^ with a difference of less than 0.2. This demonstrated that the sample size and number of terms in the models does not change even if new terms are added. It also confirmed that the models were highly significant, as shown in Table 4. There was a high correlation between the predicted values from the fitted model and the experimental data points. Above all, based on the *p*-values of the LOF, there is no significant difference between the experimental and predicted model data, which suggests the models developed have good predictions for CIP, ENRO, and COD removal.

The graphs (Figure 7), which were plotted to show the effect of the two most significant variables (pH and OLR) on the removal efficiency, vary within the determined experimental ranges by keeping one variable at a fixed level (central point level). It was found that the degradation of CIP (80–86%) will be more effective under a slightly acidic medium at a pH of 6–6.5 with an appropriate OLR (4–5 kgCOD·m^−3^·days^−1^) at a high antibiotic concentration (80–100%), as shown in Figure 7a. In the same way, in Figure 7b, the degradation of ENRO was effective under a basic medium at pH 7.5–8 with a high OLR (5–6 kgCOD·m^−3^·days^−1^). Moreover, it was found that the insignificant change of COD in the wastewater was as a result of an increase in the antibiotic concentration. Thus, an increase in the antibiotic concentration (50–100%) resulted in forming intermediate compounds, which are difficult to be degraded. In Figure 7c, within low antibiotics concentration of 30–45%, high OLR (5–6 kgCOD·m^−3^·days^−1^), and a basic medium of pH 7.5–8, COD removal of 75–83% was obtained. Using these conditions, the maximum CIP, ENRO, and COD removal efficiencies were 80%, 83%, and 73%, respectively (Figure 8). This was achieved at optimum conditions of pH (6) and OLR (2 kgCODm^−3^·days^−1^), and an antibiotic concentration of 75%. The result obtained is in agreement with Conde-Cid et al. [23], Rodríguez-López et al. [12], and Haffiez et al. [5] who reported pH as one of the most important parameters for maximizing the efficiency of pollutants from aqueous solutions. Thus pH affects the biodegradation process via the rate of the chemical reactions and generation of radicals in the process [5]. A confirmatory experiment was carried out under the optimum conditions and a desirability performance of 85% removal of the contaminant was achieved. This confirmed the accuracy and suitability of the model for its adaptability within the designed space.

This finding can be attributed to more time required to complete antibiotic degradation. Thus, the antibiotics act as barriers, which suppress the activity of the microbes in the substrate. In addition, increasing the antibiotic concentration at a low OLR can lead to a reduction in the degradation efficiency. The pH was found to be the main significant factor which led to the production of the oxidation radicals for the further degradation of the organics’ and antibiotics’ concentrations. This might be due to the antibiotic properties, such as their point of zero charge and pKa values.

## 4. Materials and Methods

### 4.1. Materials and Methods

#### 4.1.1. Reagents

Table 5 presents properties of ciprofloxacin (CIP) and enrofloxacin (ENRO). Standards were purchased from Sigma-Aldric. For pH adjustment, sulfuric acid (H_2_SO_4_) and hydrochloric acid (HCl) were purchased from Merck. All chemicals used were of analytical and HPLC grades and ultrapure water was used in the analysis. Individual stock standard solutions were prepared for the antibiotics and stored at 4 °C. Working solutions were thereafter prepared from the stock solutions.

#### 4.1.2. Analytical Method Using HPLC

Antibiotic removal was monitored using an ultrafast high-performance liquid chromatography (UHPLC) using a SPD-M20A-Photodiode Array detector (PDA) (Shimadzu), Durban, South Africa, incorporating a Gemini C-18 column (150 × 4.6 mm × 5 µm) from Phenomenex. The mobile phase was a mixture of A (water with 0.1% formic acid) and B (acetonitrile with 0.1% formic acid). A simple isocratic method was employed consisting of 15% B and a run time of 15 min [23]. The sample injection volume was 10 µL at a flowrate of 1 mL/min. The pH was measured using an Orion pH meter.

### 4.2. Wastewater and Sludge Samples

Synthetic wastewater was used in this study. It was prepared to emulate that from a slaughterhouse, as reported in a previous study by Chollom et al. [24]. The synthetic wastewater was characterized to be BOD/COD ratio of 0.40–0.53, comparable to slaughterhouse wastewater [25]. Standard method of characterizing wastewater was used to measure wastewater parameters [26]. The initial COD and BOD concentrations observed were 1499 mg/L and 674.5 mg/L, respectively. The sludge was collected as digested sludge from a local wastewater treatment plant treating slaughterhouse wastewater in South Africa. It was found to have total suspended solids (TSS) of 19.4 g/L and volatile suspended solids of (VSS) of 13.8 g/L.

#### Biodegradation

Biodegradation of antibiotics was carried out using batch experiments using 500 mL serum bottles with a working volume of 400 mL. The bottles were dosed with standard solution of the mixed compounds, and the pre-determined amounts of sludge and wastewater was added to it where necessary. The concentration of the wastewater was varied according to the required organic loading rate (OLR), as shown in Table 5. The pH of the wastewater was also adjusted, as specified by the runs understudy. To ensure that there was no interference with oxygen, the bottles were purged with nitrogen before loading and after loading. The headspace was again flushed with nitrogen. After flushing, the bottles were sealed with gas-tight silicone septa and aluminum rings with a crimping tool. The sealed bottles were incubated at 35 °C throughout the batch process time (28 days). They were shaken at a low speed of 100 rpm. The initial antibiotic concentration was 100 µg/L for the preliminary studies. The initial (1499 mg/L) and final COD was measured as an indicator of the biological activity. All the bottles used for the studies were wrapped with aluminum foil to avoid possible photolysis and photodegradation of the compounds. Two control reactors were used, one with antibiotics and the other without.

### 4.3. Experimental Design

Kinetics were evaluated using the OriginPro Software (Version 2019). Additionally, Design Expert software (10.0.3) was used to perform the design of experiments, model the data, and study the relationship between dependent and independent factors. Three independent variables selected were pH (A), OLR (B), and antibiotics concentration (C). The low, center, and high levels of each variable were designated as −1, 0, and +1, respectively, as illustrated in Table 2. These were selected based on the preliminary studies that were carried out. The OLR is a measure of the capability of the microorganism in the AD process to breakdown organic compounds present in the effluent. The dependent variables or responses were functions of removal efficiency of CIP, ENRO, and COD, as shown in Table 6.

Sorption efficiency as well as COD reduction was calculated according to Equation (6)

(6) $$\%\;reduction=\frac{\left(C_{0}-C_{t}\right)\times 100}{C_{0}}$$

where *C*_0_ and *C_t_* represent initial and final concentration of the contaminants, respectively.

## 5. Conclusions

In this study, the Box–Behnken response surface design has proven to be reliable, economical, and effective for optimization. The findings from this study show that biodegradation conditions have a significant effect on antibiotic metabolism in wastewater. The response surface plots were applied to estimate the interactive effect of three key input variables (pH, OLR, and antibiotics concentration) on the responses. The response models were developed by regression analysis (ANOVA) of the experimental data obtained from 15 runs, and their predicted results were in good agreement with the experimental values. The regression correlation presented high determination coefficient (R^2^; 0.9834–0.9875) values closer to 1, as well as high adjusted determination coefficient (Adj-R^2^) values with differences of less than 0.2 at a 95% confidence level. Among the three factors evaluated, pH was found to have a high influence on all the responses, with a positive impact on CIP and negative influence on ENRO and COD removal. In addition, the antibiotics concentration had a low impact on the responses as a factor, which ended up generating intermediate compounds, contributing to an increase of the COD content in the wastewater. Operational bottlenecks are bound to occur at high antibiotic concentrations with a low OLR because of microbial competition over nutrients to enhance degradation. The optimum conditions obtained from the numerical desirability optimization technique were pH (6), OLR (2 kg·m^−3^·days^−1^), and an antibiotic concentration of 75%, whereby a maximum removal of the contaminants with desirability of 85% was attained. Among the kinetic models examined (Origin software), the first-order model was found to be the applicable kinetic model with half-lives that ranged from 10 to 14 days for CIP and Enro degradation. This is a fact that antibiotics have the capacity to suppress microbial activity as well as agglomerate with the substrates. This study, therefore, forms the basis for minimizing the inhibitory effect of antibiotics in a wastewater setting by focusing on biodegradation and adsorption as the major route in biological systems.

## Figures and Tables

**Table 1:** ANOVA Gompertz model summary.

Antibiotics Type	ENRO	CIP
Sum of Squares (RSS)	714.3	954.7
*p*-values	<0.001	<0.001
C_t_ (mg/L)	2270 ± 37	2242 ± 62
Co (mg/L)	10 ± 16	8 ± 48
K (days^−1^)	0.0958	0.148
R^2^	0.9987	0.9805
Adj-R^2^	0.99864	0.97915

Co—initial concentration, C_t_—initial concentration, R^2^—Coefficient of regression, Adj-R^2^—Adjustable R^2^.

**Table 2:** Experiment design matrix in actual units and experimental responses.

	Input Variables			R_1_-CIP Removal		R_2_-ENRO Removal		R_3_-COD Removal	
Run	A:pH	B:OLR	C:ANT Con	Exp	Pred	Exp	Pred	Exp	Pred
		(kg·m^−3^·Days^−1^)	%	%	%	%	%	%	%
1	7	2	10	69	70	68	68	80	80
2	7	6	10	68	68	66	67	82	82
3	7	4	55	77	78	74	76	76	75
4	8	2	55	75	74	76	74	75	74
5	7	4	55	78	78	75	76	74	75
6	6	2	55	76	77	78	78	76	77
7	8	4	10	69	69	70	70	80	80
8	7	2	100	83	83	86	85	68	68
9	7	4	55	78	78	74	76	76	75
10	6	4	10	69	68	65	64	83	82
11	8	4	100	79	80	85	86	67	68
12	8	6	55	75	75	84	81	78	77
13	6	4	100	86	86	86	85	68	68
14	7	6	100	85	84	85	86	69	69
15	6	6	55	75	76	73	71	75	76

Exp—Experimental results, Pred—Predicted results, R—response.

**Table 3:** Model coefficient estimation.

CIP			ENRO			COD		
Intercept	Coefficient Estimate	Standard Error	Intercept	Coefficient Estimate	Standard Error	Intercept	Coefficient Estimate	Standard Error
	77.67	0.715		74.54	0.58		75.33	0.75
A-pH	−1.00	0.44	A-pH	1.63	0.43	A-pH	−0.25	0.46
B-OLR	0.63	0.46	B-OLR	0.0	0.43	B-OLR	0.63	0.46
C-ANT	−6.63	0.46	C-ANT	9.13	0.43	C-ANT	−6.63	0.46
AB	1.00	0.65	AB	3.25	0.6	AB	1.00	0.65
AC	0.50	0.65	AC	−1.50	0.61	AC	0.50	0.65
BC	−0.25	0.65	BC	0.25	0.61	BC	−0.25	0.65
A^2^	0.21	0.68	A^2^	1.81	0.63	A^2^	0.21	0.68
B^2^	0.46	0.68	B^2^	1.56	0.63	B^2^	0.46	0.68
C^2^	−1.04	0.68				C^2^	−1.04	0.68

**Table 4:** Model fit statistics and regression analysis.

	R_1_-CIP Removal	R_2_-ENRO Removal	R_3_-COD Removal		R_1_-CIP Removal	R_2_-ENRO Removal	R_3_-COD Removal
Source	*p*-Value	*p*-Value	*p*-Value	Fit Statistics			
**Model**	0.0013	<0.0001	0.0006	**R^2^**	0.9834	0.9827	0.9875
A-pH	0.0012	0.0011	0.0012	**Adjusted R^2^**	0.9735	0.9748	0.9669
B-OLR	0.0312	0.0321	0.0001	**Predicted R^2^**	0.9642	0.9606	0.9578
C-ANT (%)	<0.0001	<0.0001	<0.0001	**Adeq Precision**	17.8033	16.7472	13.8056
AB	0.0238	0.0088	0.0031	**Std. Dev.**	1.24	1.89	1.30
AC	0.0457	0.0211	0.0368	**Mean**	76.13	76.33	75.13
BC	0.0058	0.0281	0.0099	**C.V.%**	1.63	2.48	1.73
A^2^	0.0701		0.0731				
B^2^	0.0274		0.0071				
C^2^	0.035		0.0087				

**Table 5:** Physicochemical properties of Ciprofloxacin and Enrofloxacin antibiotics [17].

Antibiotics	MW	Class	Structure	pKa 1 and 2
Ciprofloxacin	332.3	Fluoroquin-olone	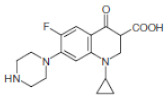	6.68/8.63
Enrofloxacin	359.4	Fluoroquin-olone	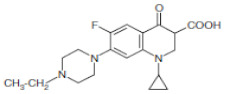	5.86/8.24

MW—molecular weight.

**Table 6:** Experimental range and levels of the independent variables.

Input Variables	Values		
Levels (coded)	−1	0	1
A: pH	6	7	8
B: OLR (kgCOD·m^−3^·days^−1^)	2	4	6
C: ANT Concent (%)	10	55	100

## Data Availability

Not applicable.

## Sample Availability

Samples of the compounds are not available from the authors.
